# CircERCC6 Positively Regulates the Induced Activation of SHF Stem Cells in Cashmere Goats via the miR-412-3p/BNC2 Axis in an m^6^A-Dependent Manner

**DOI:** 10.3390/ani14020187

**Published:** 2024-01-05

**Authors:** Qi Zhang, Yixing Fan, Man Bai, Yubo Zhu, Zeying Wang, Jincheng Shen, Ruqing Xu, Wenxin Zheng, Wenlin Bai

**Affiliations:** 1 College of Animal Science & Veterinary Medicine, Shenyang Agricultural University, Shenyang 110866, China; 2 State Key Laboratory for Herbivorous Livestock Genetic Improvement and Germplasm Innovation of Ministry of Science and Technology and Xinjiang Uygur Autonomous Region, Urumqi 830011, China; 3 Xinjiang Academy of Animal Sciences, Urumqi 830011, China

**Keywords:** CircERCC6, *N*^6^-Methyladenosine, SHF stem cells, induced activation, cashmere goats

## Abstract

**Simple Summary:**

Cashmere goats have been widely raised in northern China, and they have great economic importance to local farmers and herders. The cashmere is one of the main uses of cashmere goats, and it is produced from the secondary hair follicles (SHFs) of cashmere goats. The biological event of induced activation of SHF stem cells by the dermal papilla cell-derived signals is essential to the anagen initiation and reconstruction of cashmere goat SHFs; however, its precise molecular mechanism is still unknown. In the present investigation, the expression level of m^6^A-modified circERCC6 (m^6^A-circERCC6) was found to be significantly higher at anagen SHF bulge than that of telogen. Further, we demonstrated that the circERCC6 positively regulates the induced activation of SHF stem-cells via the miR-412-3p/BNC2 axis in cashmere goats, and the m^6^A modification of circERCC6 is required for its function exertion. The obtained results provided a novel molecular basis for uncovering the intrinsic regulatory mechanisms of the induced activation process of SHF stem cells in cashmere goats. Also, the results will provide significant information for improving the SHF regeneration ability of cashmere goats with the growth of cashmere fibers.

**Abstract:**

The cashmere, a kind of nature protein fiber, is one of the main use of cashmere goats. The induced activation of secondary hair follicle (SHF) stem cells by the dermal papilla cell-derived signals is a key biological process for the morphogenesis and growth of cashmere fiber in cashmere goats. Previously, the circRNA-ERCC6 (circERCC6) was identified from cashmere goat SHFs; however, its biological significance is unclear in the SHF physiology process of cashmere goats. In this study, we found that circERCC6 exhibited significantly higher expression at anagen SHF bulge compared with the counterpart of telogen and harbored three m^6^A modified sites (named m^6^A-685, m^6^A-862, and m^6^A-995) through methylation immunoprecipitation using a real-time quantitative polymerase chain reaction (Me-RIP-qPCR) technique. The knockdown experiments of circERCC6 in SHF stem cells showed that circERCC6 positively regulates the induced activation of SHF stem cells in cashmere goats. Through a dual-luciferase reporter assay, we demonstrated that m^6^A-modified circERCC6 (m^6^A-circERCC6) sponged miR-412-3p to upregulate the expression of BNC2 mRNA in SHFstem cells. Through m^6^A-deficient mutant assay in circERCC6 knockdown SHF stem cells, we further showed that m^6^A modification within circERCC6 is required to mediate the miR-412-3p/BNC2 axis to finally promote the proper induced activation of SHF stem cells in cashmere goats.

## 1. Introduction

Cashmere goats have been widely raised in northern China, and they have great economic importance to local farmers and herders [1,2]. There are two different fiber types in the fleece of cashmere goats, i.e., the coarse hair and the fine cashmere [3]. As is well known, the cashmere is the main use of cashmere goats. The cashmere is produced from the secondary hair follicles (SHFs)—dynamic mini-organs in skin tissue of cashmere goats—and its morphogenesis along with subsequent growth is regulated by the activity of SHFs. The induced activation of SHF stem cells by the dermal papilla cell-derived signals is essential to the anagen initiation and reconstruction of cashmere goat SHFs, which is closely related to the cashmere growth and production per growth cycle and even influences on the SHF transition from telogen to anagen [4]. As is known, the induced activation of hair follicle stem cells is a complicated biological process that is coordinately regulated by many regulatory factors including some non-coding RNA molecules [5,6,7,8].

Circular RNA (circRNA), a kind of non-coding RNAs, is characterized by its unique structure—a covalently closed loop without free 5′ and 3′ ends. Over the past few years, circRNAs were progressively identified in the skin tissue and SHFs of cashmere goats [9,10]. Of them, some were revealed to exhibit significantly differential expression between anagen and telogen of cashmere goat SHFs [6,10], which implies that circRNAs may be essentially implicated in the SHF physiological process of cashmere goats, like SHF regeneration, development via morphogenesis, and growth of cashmere fiber. An outstanding example exists in the differentiation of cashmere goat SHF stem cells, where several circRNAs were found to positively regulated the differentiation process of SHF stem cells via miRNA-mediated pathways in cashmere goats, including circRNAs-1926, -0100, and -1967 [2,9,11].

In recent years, some circRNAs are revealed to contain extensive *N*^6^-Methyladenosine (m^6^A) modification sites that essentially participate in the functional exertion of the circRNAs in an m^6^A-dependent manner [12,13]. In humans, it was demonstrated that m^6^A modification of circ-ARL3 contributes Hepatitis-B-virus-associated hepatocellular carcinoma via miR-1305-mediated mechanisms [14]. Also, it was reported that m^6^A modification within circRNA molecules was heavily implicated in the biosynthesis circRNA itself and its stable and long-lasting protein production [15]. More recently, Hui and colleagues identified fifteen m^6^A modification circRNAs (m^6^A-circRNAs) from the skin tissue of cashmere goats, of which six m^6^A-circRNAs were found to exhibit significantly higher expression in anagen skin tissue in comparison to the those of telogen [16]. Moreover, the m^6^A-circRNA-ZNF638 was proved to promote the induced activation of SHF stem cells in cashmere goats via an m^6^A-dependent mechanism [6]. 

The circRNA-ERCC6 (circERCC6) was recently identified from cashmere goat SHFs with the ERCC6 being its host gene, and it exhibited significantly higher expression at anagen SHFs in comparison to the counterpart at telogen [10]. However, its biological significance is unclear in the SHF physiology process of cashmere goats. The main purposes of this study were as follows: (1) Investigate the effects of circERCC6 on the induced activation of SHF stem cells in cashmere goats; (2) Explore the potential molecular mechanisms of circERCC6 regulating the induced activation of SHF stem cells. The results from this study would provide a novel treating target for artificially regulating SHF regeneration and cashmere fiber growth to enhance the cashmere yield and quality in cashmere goats. 

## 2. Materials and Methods

### 2.1. Collection of Skin Tissue, Preparation, and Extraction of Total RNA

In this investigation, we carried out all experiments according to the experiment protocol guidelines (No. 2023030208) that had been reviewed and approved by the Experimental Animal Ethics and Welfare Committee of Shenyang Agricultural University (Shenyang, China). Six goats (adult and female) were sampled from the Liaoning cashmere breed without traceable genetic relationships. The skin tissue from each goat was collected according to the described assay by Zhu and colleagues [2]. In brief, the sampled goats were subjected to local anesthesia (posterior margin location of scapula), and the skin tissue (about 1 cm^2^) was collected using a sterilized surgical blade. After soaking and cleaning with 75% alcohol, the skin tissue was cut into pieces of 5 mm^2^. After washing three times using, the tissue samples were digested with 0.25% dispase II at 4 °C overnight. Under a stereo microscope, we separated the SHFs from skin tissue using a sterilized scalpel and disposable syringe needle. Finally, the SHF bulge section was separated according to the described assay by Ohyama and Kobayashi [17]. Using the RNAiso reagent kit, the total RNA was extracted according to the manufacturer’s instructions (TaKaRa, Dalian, China). 

### 2.2. Characteristic Analysis of Cashmere Goat circERCC6 Sequence

Here, the analyzed circERCC6 was previously identified from the skin tissue of cashmere goats [10]. We defined the source of circERCC6 transcription via aligning the linearized sequence of its spliced length into goat genome sequences deposited in the GenBank database (https://www.ncbi.nlm.nih.gov (accessed on 8 February 2023), ARS1 version). Two procedures, miRDB (http://www.mirdb.org (accessed on 11 March 2023)) and RNAhybrid (https://bibiserv.cebitec.uni-bielefeld.de/rnahybrid (accessed on 11 March 2023)), were used to predict the potential binding sites of miRNAs in a circERCC6 sequence. The miRNAsong database (https://www2.med.muni.cz/histology/miRNAsong/index.php (accessed on 11 March 2023)) was utilized to retrieve each mature miRNA sequence. The analysis of potential m^6^A modification sites in circERCC6 was performed using the SRAMP program (http://www.cuilab.cn/sramp (accessed on 22 March 2023)). 

### 2.3. Analysis of Overexpression and Knockdown of circERCC6 in SHF-Stem Cells

The overexpression or knockdown of circERCC6 was performed in SHF stem cells of cashmere goats that were stored in our laboratory. The overexpression vectors of circERCC6 (or its mutants) were generated following the assay described in a previous investigation [9], where the circERCC6 mutants were generated using the QuikChange Lightning Multi Site-Directed Mutagenesis Kit (Agilent, Technologies, Santa Clara, CA, USA). In short, the pcDNA3.1 (+) circRNA mini-vector (Addgene, Cambridge, MA, USA) was used to construct the overexpression vector of circERCC6 or its mutant. The recombinant pcDNA3.1 (+) circERCC6 (or its mutant) was transiently transfected into the passage 3 SHF stem cells using the Lipofectamine 3000 (Invitrogen, Carlsbad, CA, USA). For the analysis of circERCC6 knockdown in SHF stem cells, three siRNAs (si-circERCC6-1, si-circERCC6-2, and si-circERCC6-3) were designed according the back splice sequence of circERCC6. The designed sequences of three siRNAs to circERCC6 aresi-circERCC6-1: 5′-CCACGCTCGCTCCCGGGCTGAC-3′, si-circERCC6-2: 5′-CCGGG- CTGACTTTCTGAAAAGC-3′, and si-circERCC6-3: 5′-TCGCTCCCGGGCTGACTTTCT- G-3′. The passage 3 SHF stem cells were transiently transfected, respectively, using the three siRNAs through the use of the siRNAs Lipofectamine RNAiMAX kits (Invitrogen, Shanghai, China). 

The transfected SHF stem cells were subjected to non-contacting co-culture with dermal papilla cells (DPCs) of cashmere goats (stored in our laboratory) for induced activation in a transwell device as described in a previous investigation [5]. In short, the passage 3 SHF stem cells were seeded on six-well plates, in which a transwell insert seeded with passage 3 DPCs was added for co-culture of the two types of cells: SHF stem cells and DPCs. Co-culture of the cells was performed in fresh DMEM/F12 medium (Hyclone, Logan, UT, USA) supplemented with 10% fetal bovine serum. The incubator was set as 37 °C with 5% CO_2_. The medium was changed every 2 days.

### 2.4. Designing of RT-qPCR Primers and the Reaction Assay

For circERCC6, the primers (divergent primers) were designed in the previous investigation by Shen and colleagues [10]. For the mRNA detection of related genes and the Me-RIP-qPCR of circERCC6, we designed convergent primer pairs through the use of the Primer Premier 5.0 procedure (http://www.premierbiosoft.com (accessed on 4 April 2023)). In addition, for the detection of each miRNA, the sense primer was designed according to the mature miRNA sequence and the corresponding anti-sense primer of each analyzed miRNA that was provided along with the kits was universal (TaKaRa, Dalian, China). The U6 primer pair was designed in the previous investigation by Han and colleagues [18]. All primers used in this study are listed in Table 1. 

Using the M-MuLV cDNA synthesis kit (Sangon, Shanghai, China), we used random primers to synthesize the first-strand cDNAs for analyzing the expression of circERCC6 and the implicated gene mRNAs. Whereas, the One-Step PrimeScript microRNA cDNA synthesis kit (TaKaRa, Dalian, China) was used to analyze the miRNA expression. In a final volume of 25 μL, the qPCR reactions were performed where the reaction system was comprised of Green Premix Ex Taq II of 12.5 μL TB (Tli RNaseH Plus, TaKaRa, Dalian, Chnia), the first-strand cDNA solution of 2.0 μL, each primer of 1.0 μL (10 μM), and ddH_2_O water of 8.5 μL. In qPCR reactions, the thermal cycling parameters were set as a single cycle of 95 °C for 3 min, followed by 40 cycles of 95 °C for 5 s, 53–59 °C (Table 1) for 30 s, and 72 °C for 30 s. The m6A relative abundance of circERCC6 was normalized to the input. Whereas, the GAPDH and U6 genes were used as internal references for the expression of analyzed gene mRNAs and miR-412-3p, respectively, with the relative expression calculated by using the 2^−∆∆Ct^ method [19]. 

### 2.5. Methylation Immunoprecipitation of circERCC6 (Me-RIP) Assay

We performed the Me-RIP analysis of circERCC6 following the methods by Chen and colleagues [20]. In brief, the extracted total RNA (100 μg) was treated with Ribonuclease R (RNase R, Geneseed, Guangzhou, China), followed by a concentration using the Monarch RNA Cleanup Kit (NEB, Ipswich, MA, USA). Then, we fragmented the concentrated RNA using the NEBNext Magnesium RNA Fragmentation Module (NEB, Ipswich, MA, USA) at 94 °C for 3 min, followed by further concentration using the Monarch RNA Cleanup Kit (NEB, Ipswich, MA, USA). Of the fragmented RNA product, 2 μg RNA was stored as an input control. The half-fragmented RNA product was subjected to incubation (4 °C for 4 h) with 2 μg anti- m6A antibody (Synaptic Systems, Gottingen, Germany) or IgG (Cell Signaling Technology, Danvers, MA, USA). Subsequently, the Dynabeads Protein A (Thermo Scientific, Rockford, IL, USA) was pre-washed, followed by incubation with the complex of RNA and antibody (at 4 °C for 2 h). Finally, we isolated the RNA using the RNAiso kit (TaKaRa, Dalian, China). The relative abundance of circERCC6 was analyzed by using the RT-qPCR technique and was normalized to the input control [20].

### 2.6. Dual-Luciferase Reporter Assays

Here, we performed the dual-luciferase reporter analysis according to the methods described by Yu and colleagues [21]. In short, to confirm the specifically binding of circERCC6 with miR-412-3p in SHF stem cells, the luciferase reporters were generated by ligating the circERCC6 (circERCC6-WT) or the corresponding mutant (circERCC6-MUT) to pGL3-basic vectors (Promega, Madison, WI, USA). Using the the QuikChange Lightning Multi Site-Directed Mutagenesis Kit (Agilent, Technologies, Santa Clara, CA, USA), we generated the circERCC6 mutant (circERCC6-MUT) within which the putative binding sites of the miR-412-3p seed region were mutated with their corresponding complementary bases. Similarly, to define the specific binding of the *BNC2* mRNA 3′-untranslated region (3′-UTR) with miR-412-3p in SHF stem cells, we amplified the 3′-UTR fragment of cashmere goat *BNC2* mRNA, which harbored the putative binding site of miR-412-3p and further ligated to the pGL3 basic vector (Promega, Madison, WI, USA). Subsequently, the generated reporter vectors were, respectively, transfected into the passage 3 SHF stem cells using the Lipofectamine 2000 (Invitrogen, Carlsbad, CA, USA). The transfected SHF stem cells were cultured as described above. After 48 h, the cells were harvested, and the signals of luciferase activities were detected using the Dual-Luciferase Reporter Assay System (Promega, Madison, WI, USA). Finally, we calculated the relative luciferase activity ratio of firefly luciferase activity to renilla luciferase activity to eliminate the potential deviation from transfection efficiency differences among the tested samples. 

### 2.7. Data Statistical Analysis

The statistical analysis of all obtained data was performed using the SPSS 17.0 procedure (SPSS Inc., Chicago, IL, USA). The analyzed results were presented using the GraphPad Prism software (Version 8.3.0). The difference between two groups was compared using Student’s *t*-test. An obtained *p* value less than 0.05, 0.01, and 0.001 was defined as a significant, highly significant, and extremely significance differences, respectively. 

## 3. Results and Discussion

### 3.1. Molecular Characterization Analysis of SHF circERCC6 in Cashmere Goats

**Figure 1:**
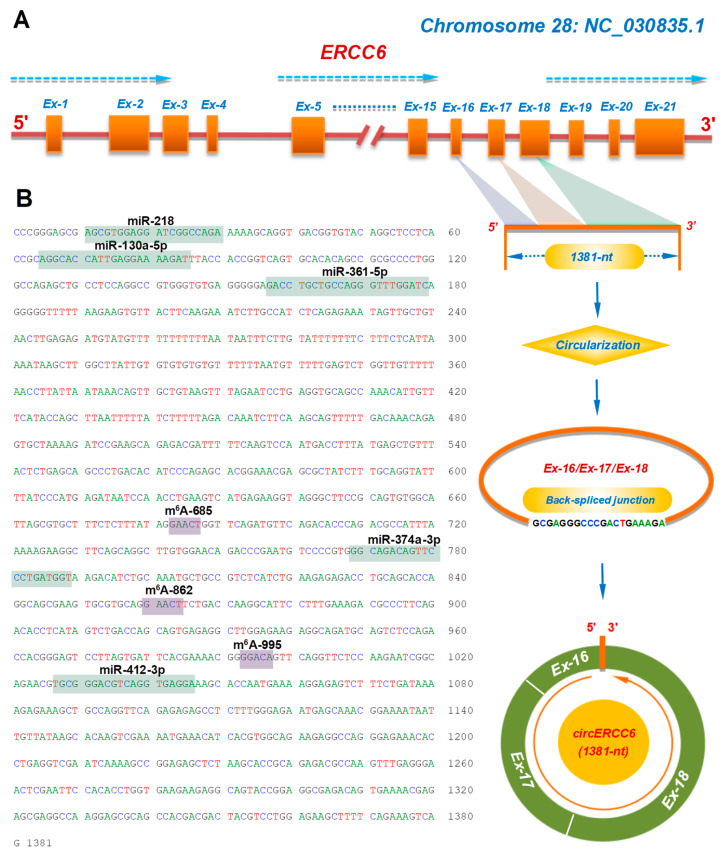
The transcription source and molecular characteristics of circERCC6 in cashmere goats: (**A**) Overall diagram of the host source of circERCC6 with its reverse splicing pattern and the splicing size of 1381-nt. (**B**) The cDNA sequence of circERCC6 with the potential binding sites of miRNAs and m^6^A modification sites. The potential binding sites of five miRNAs within the circERCC6 sequence were indicated by green shading with each miRNA name including miR-218, miR-130a-5p, miR-361-5p, miR-374a-3p, and miR-412-3p. Also, the potential m^6^A modification sites were indicated by purple shading with each name given including m^6^A-374, m^6^A-685, m^6^A-862, and m^6^A-995.

The circERCC6, for the first time, was identified from SHFs (1381-nt in spliced length) of cashmere goats [10]. Through aligning its linearized sequence in the goat genome, we found that it was formed by a reverse splicing of exons-16, -17, and -18 of the goat ERCC6 gene on chromosome 28 (Figure 1A). 

It is widely accepted that circRNAs can sequester miRNAs to exert their functional significance in regulating the expression of corresponding target genes [22]. Based on in-silicon prediction, we found that circERCC6 harbored putative binding sites of several miRNAs, including miR-218, miR-130a-5p, miR-361-5p, miR-374a-3p, and miR-412-3p (Figure 1B), which suggests that a miRNA-mediated mechanism may be implicated in the functional roles of circERCC6 in SHF physiological processes of cashmere goats. Meanwhile, we also noted that three potential m^6^A modification sites exist within the circERCC6 sequence with the m^6^A motif GA/GACU/A (Figure 1B) that is consistent with the revealed m^6^A motif RRACH (R = A or G; H = A, C or U) in linear RNAs [23]. Thus, it is possible that three m^6^A sites are significantly implicated in the function exertion of circERCC6, although the existence of these three m^6^A sites needs be further confirmed in the SHFs of cashmere goats.

### 3.2. Expression Patterns of circERCC6 in SHF Bulge of Cashmere Goats and Its Roles in Regulating the Induced Activation of SHF Stem Cells

**Figure 2:**
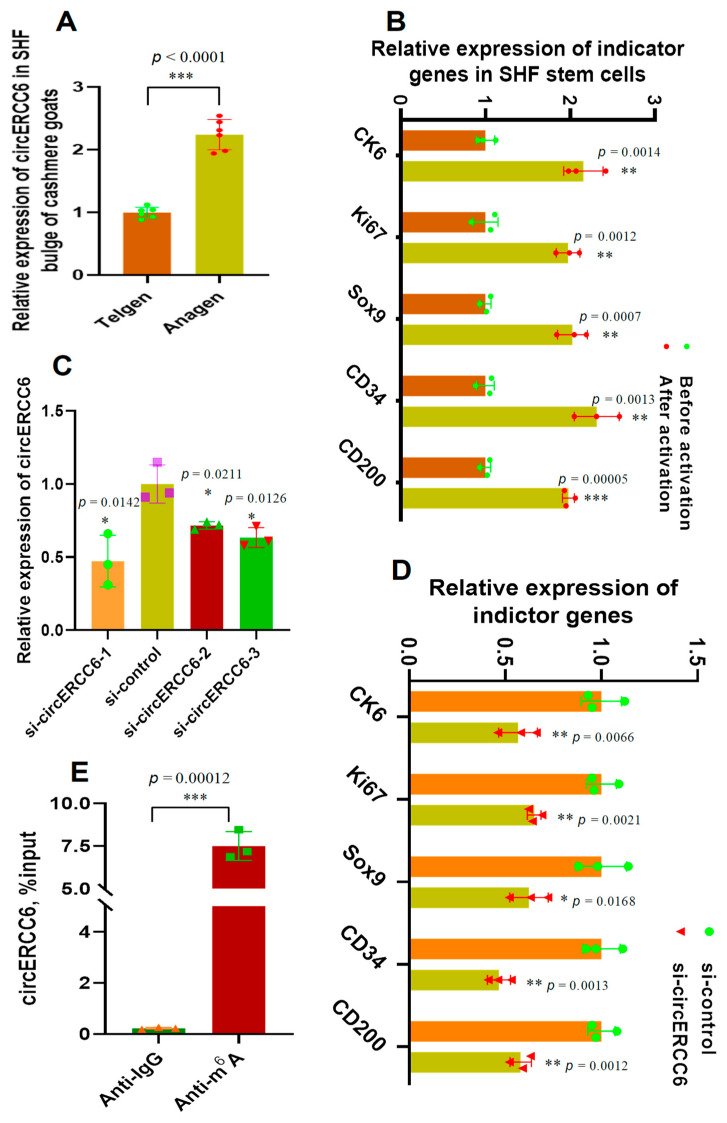
The expression pattern of circERCC6 in the SHF bugle of cashmere goats with its effect on the induced activation of SHF stem cells in cashmere goats: (**A**) Relative expression of circERCC6 at anagen and telogen SHF bugles of cashmere goats. (**B**) Relative expression of indicator genes before and after induced activation of SHF stem cells. (**C**) Knockdown efficiency of 5′-CCACGCTCGCTCCCGGGCTGAC-3′, si-circERCC6-2: 5′-CCGGGCTGACTTTCTGAAAAGC-3′, and si-circERCC6-3: 5′-TCGCTCCCGGGCTGACTTTCTG-3′ to circERCC6 in SHF SCs of cashmere goats. (**D**) Knockdown of circERCC6 resulted in significant decreasing expression of the indictor genes in SHF stem cells. (**E**) Enrichment analysis of m^6^A modification sites within circERCC6 in SHF stem cells where the percentage of the input is shown. The “*”, “**”, and “***” indicate significant difference with *p* < 0.05, *p* < 0.01, and *p* < 0.001, respectively.

In order to uncover the expression characteristics of circERCC6 in the SHF bugle of cashmere goats, we investigated its relative expression at both anagen and telogen SHF bulges. The obtained results were presented in Figure 2A. We found that a significantly higher expression of circERCC6 at anagen SHF bulges was recorded compared with the counterpart of telogen. In fact, during anagen, the bulge SHF stem cells are under an exuberant period of induced activation [4], which was also supported well by the investigated results in a previous publication [6]. Thus, it is reasonable to assume that circERCC6 may play certain roles in regulating the induced activation process of SHF stem cells in cashmere goats. 

To verify this assumption, we firstly tested the expression changes of several indicator genes (upon induced activation of SHF-stem cells, including CK6, Ki67, Sox9, CD34, and CD200) in SHF stem cells before and after induction activation. Further, we performed a knockdown experiment of circERCC6 in SHF stem cells using a siRNA inference technique. As expected, the tested indicator genes had significantly higher relative expression in SHF stem cells after induced activation in comparison to the counterpart of previously induced activation (Figure 2B). The efficiency analysis of the designed three siRNAs (si-circERCC6-1, si-circERCC6-2, and si-circERCC6-3) showed that si-circERCC6-1 had better knockdown efficiency to circERCC6 in SHF stem cells than si-circERCC6-2 and si-circERCC6-3 (Figure 2C). Thus, the si-circERCC6-1 was used in further knockdown experiments for circERCC6. As recorded in Figure 2D, the si-circERCC6-mediated knockdown of circERCC6 led to a significant decrease in the expression of the tested indicator genes in SHF stem cells compared with the counterparts of the si-control group (Figure 2D). Thus, it can be suggested that circERCC6 might be significantly implicated in promoting the induced activation process of SHF stem cells with certain potential mechanisms. 

### 3.3. The m^6^A Modifications of circERCC6 Are Significantly Involved in the Induced Activation of SHF-Stem Cells

**Figure 3:**
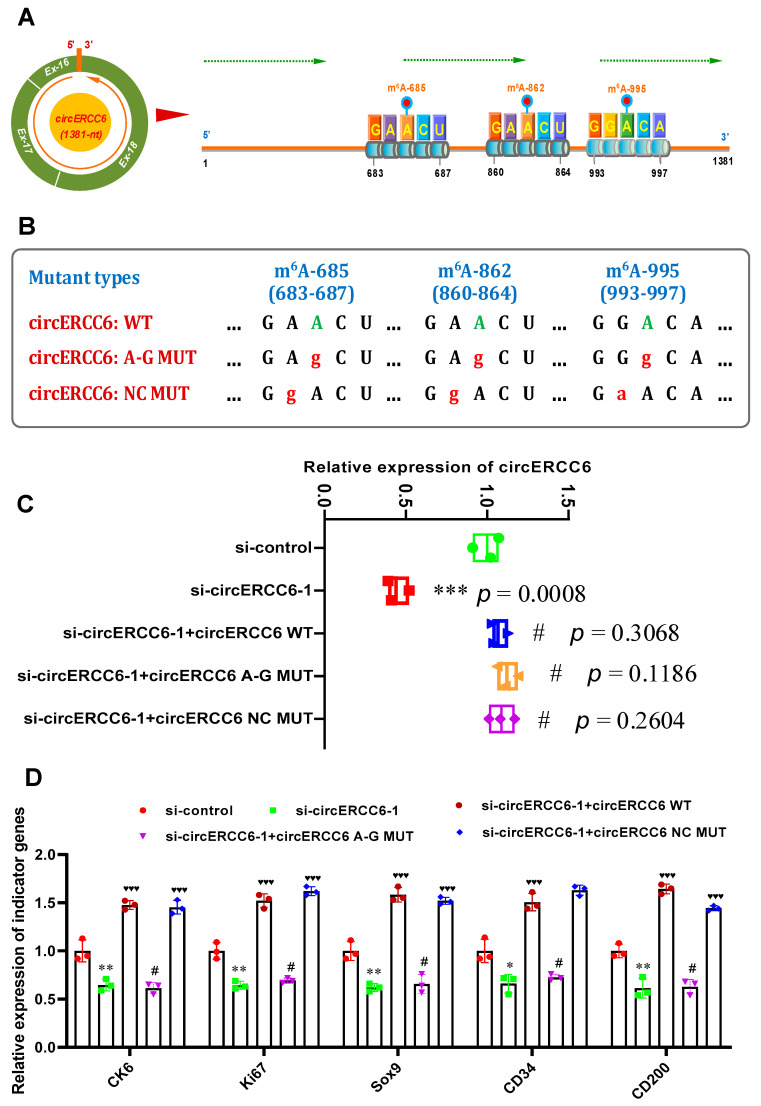
CircERCC6 contributes the induced activation of SHF stem cells under m^6^A–dependent manner: (**A**) Overall diagram of m^6^A sites in circERCC6 of cashmere goat SHFs including m^6^A–374, m^6^A–685, m^6^A–862, and m^6^A–995. (**B**) The construction strategies for circERCC6 mutants against its m^6^A sites. circERCC6:WT = wild type of circERCC6, circERCC6:A–G MUT = circERCC6 mutant against the m^6^A sites, and circERCC6:NC MUT = circERCC6 negative control mutant against the m^6^A sites. (**C**) The effects of m^6^A site mutation on circERCC6 expression in SHF stem cells with circERCC6 knockdown. The “***” indicates significant difference in comparison to the si-control group with *p* < 0.001, and the “#” indicates no significant difference in comparison to si-control group with *p* > 0.05. (**D**) The effects of m^6^A site mutation on the expression of indictor genes in SHF stem cells with circERCC6 knockdown. The “*” and “**” indicate significant difference in comparison to the si-control cell group with *p* < 0.05 and *p* < 0.01, respectively. The “♥♥♥” indicates significant difference in comparison to the circERCC6-1 cell group with *p* < 0.001. The “#” indicates no significant difference in comparison to the si-circERCC6-1 group with *p* > 0.05.

In previous investigations, it was demonstrated that the m^6^A modifications were essentially involved in the exertion of biological functions of m^6^A modified circRNAs [6,12,13]. In this study, as shown in Figure 1B, three potential m^6^A modified sites were harbored within the circERC6 sequence, and they were named m^6^A-685, m^6^A-862, and m^6^A-995, respectively (Figure 1B). Interestingly, the three potential m^6^A-modified sites of circERC6 were confirmed in SHF stem cells of cashmere goats via conducting a Me-RIP analysis (Figure 2E). These results drive us to ask whether the revealed role of circERCC6 in positively regulating the induced activation of SHF stem cells might be realized in a m^6^A-mediated manner. Thus, pointing at the confirmed three m^6^A-modification sites, m^6^A-685, m^6^A-862, and m^6^A-995 (Figure 3A), we generated the circERCC6 overexpression vectors (wild type, WT) and the corresponding overexpression mutation vectors (mutant type, MUT) within which the A685, A862, and A995 in the m^6^A motif RRACH were replaced with G685, G862, and G995 (circERCC6 m^6^A-deficient A-G mutant, circERCC6 A-G MUT), respectively (Figure 3B). Also, we generated the circERCC6 negative control mutant (circERCC6 m^6^A-decorated mutant, circERCC6 NC MUT), where the A684, A861, and G994 within the m^6^A motifs RRACH were replaced with G684, G861 and A994, respectively (Figure 3B). 

In circERCC6-knockdown SHF stem cells, we overexpressed circERCC6 (WT), the circERCC6 m^6^A-deficient A–G mutant (circERCC6: A-G MUT), or circERCC6 m^6^A-decorated mutant (circERCC6 NC MUT), to assess the possible roles of m^6^A modification in the functional exertion of circERCC6 in SHF stem cells via induced activation assays in vitro. As shown in Figure 3C, the introduction of mutations did not result in a significant difference in the circERCC6 expression in SHF stem cells; meanwhile, we also found that the decreasing expression of circERCC6 in circERCC6-knockdown SHF stem cells was restored via the transfection of circERCC6 vectors (WT) or its mutants (circERCC6: A-G MUT, or circERCC6: NC MUT) (Figure 3C). 

Subsequently, we tested the expression of the indicator genes (regarding the induced activation of SHF stem cells) in the differently treated cell lines. As shown in Figure 3D, the ‘si-circERCC6-1 + circERCC6’ cell lines were significantly higher in the expression of the indicator genes, but not the ‘si-circERCC6-1 + circERCC6 A-G MUT’ in comparison to the ‘si-circERCC6-1’ cell lines (Figure 3D). Here, we can determine that the observed expression changes in the indicator genes between two different cell lines (‘si-circERCC6-1 + circERCC6 WT’ and ‘si-circERCC6-1 + circERCC6 A-G MUT’) are not correlated with the expression abundance of circERCC6 in the analyzed cell lines, because no significant difference in circERCC6 expression was recorded between‘si-circERCC6-1 + circERCC6 WT’ and ‘si-circERCC6-1 + circERCC6 A-G MUT’cell lines (Figure 3C). As expected, we also noted that the circERCC6 m^6^A-decorated mutant (circERCC6 NC MUT) restored the expression level of the analyzed indicator genes of circRNA-ZNF638-deficient cell lines (Figure 3D). Taken together, it can be inferred that The m^6^A modifications of circERCC6 are significantly involved in the induced activation of SHF-stem cells. This revealed that the functional mechanism of m^6^A modification of circERCC6 in SHF stem cells is highly similar with those reported in m^6^A modification of linc1281 in mouse embryonic stem cells [24] and circRNA-ZFN638 in goat SHF stem cells [6].

### 3.4. CircERCC6 Sponges miR-412-3p and May Negatively Regulate Its Expression in SHF Stem Cells

**Figure 4:**
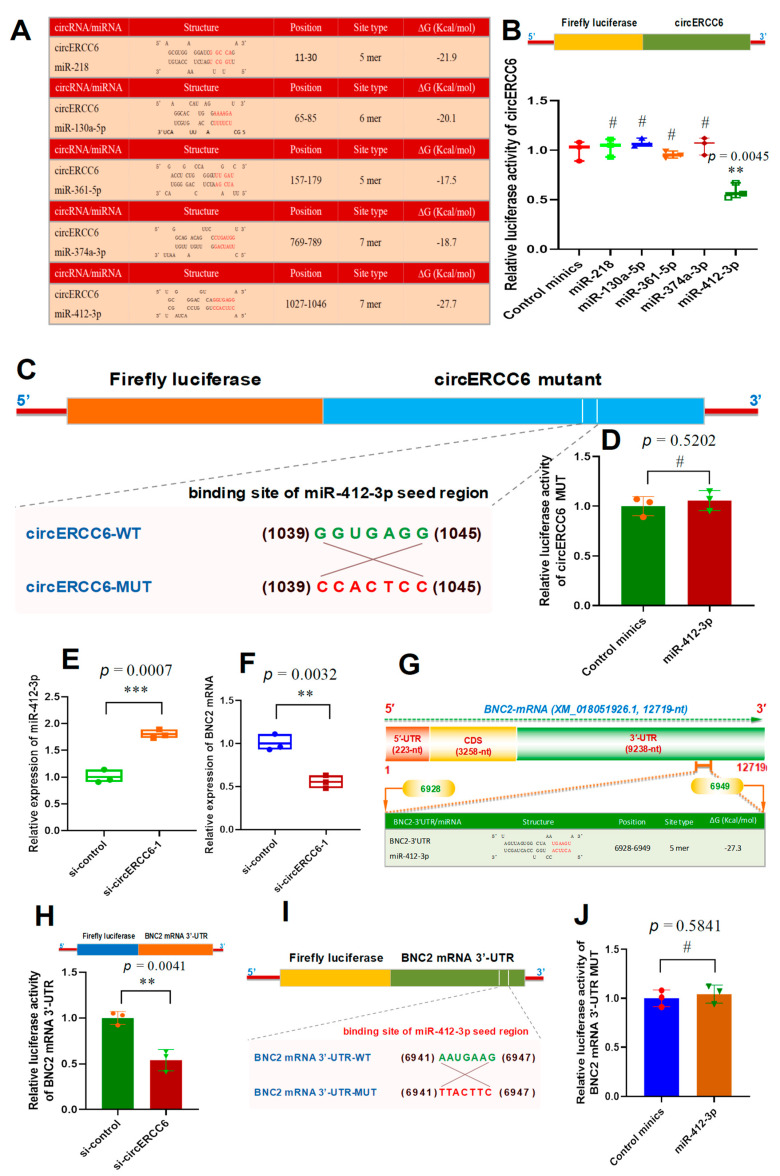
The circERCC6 serves as a miR-412-3p sponge to up-regulate the BNC2 expression SHF stem cells. (**A**) The prediction of potential miRNAs binding with circERCC6 including miR-218, miR-130a-5p, miR-361-5p, miR-374a-3p, and miR-412-3p. (**B**) Relative luciferase activities of reporters containing circERCC6 in SHF stem cells after 48 h co-transfection with the different miRNA mimics. (**C**) The construction strategies of the circERCC6 mutant (circERCC6-MUT) for the binding sites of miR-412-3p in circERCC6 sequence. (**D**) Relative luciferase activities of reporters containing the circERCC6 mutant (circERCC6 MUT) in SHF stem cells after 48 h co-transfection with the miR-412-3p mimics or control mimics. (**E**) Relative expression of miR-412-3p in SHF stem cells with circERCC6 knockdown. (**F**) Relative expression of BNC2 in SHF stem cells with circERCC6 knockdown. (**G**) A diagram of goat BNC2 mRNA accompanying the prediction of binding sites of miR-412-3p in the 3′-UTR of BNC2 mRNA. The nucleotide positions are defined based on the goat BNC2 mRNA sequence with accession number XM_018,051,926.1 at NCBI (https://www.ncbi.nlm.nih.gov (accessed on 8 Febuary 2023)). (**H**) Relative luciferase activities of reporters containing BNC2 mRNA 3′-UTR in SHF stem cells with circERCC6 knockdown. (**I**) The construction strategies of BNC2 mRNA-3′UTR mutant (BNC2 mRNA-3′UTR-MUT) for the miR-412-3p binding sites. (**J**) Relative luciferase activities of reporters containing BNC2 mRNA-3′UTR mutant (BNC2 mRNA-3′UTR-MUT) in SHF stem cells after 48 h co-transfection with the miR-412-3p mimics or control mimics. The “**” and “***” indicate significant differences with *p* < 0.01 and *p* < 0.001, respectively. The “#” indicates no significant difference with *p* > 0.05.

Increasing lines of evidence confirmed that circRNAs can sponge miRNAs to further regulate the expression level of their target genes at the post-transcriptional level [6,11,25,26]. Thus, we performed a bioinformatics analysis to screen the potential binding miRNAs within circERCC6 sequence. As shown in Figure 4A, several miRNAs potentially target the circERCC6 at different binding regions including miR-218, miR-130a-5p, miR-361-5p, miR-374a-3p, and miR-412-3p (Figure 4A). To define which miRNAs were sponged by circERCC6, we generated a luciferase reporter containing the linearized sequence of circERCC6 in the downstream of the firefly luciferase gene (Figure 4B). Subsequently, we co-transfected the generated reporters into SHF stem cells, along with single candidate miRNA mimics, respectively. The obtained results indicated that the significantly decreasing luciferase activities of circERCC6 reporters were recorded in only the miR-412-3p co-transfected group but not in the other miRNA (miR-218, miR-130a-5p, miR-361-5p, and miR-374a-3p) co-transfected groups (Figure 4B). To further verify this binding of miR-412-3p and circERCC6, we also generated circERCC6 mutation luciferase reporters (circERCC6 mutant) that contained an 7 nt long anti-sense mismatch at the seed target site of miR-412-3p (Figure 4C). After co-transfection with miR-412-3p, we noted that mutation circERCC6 reporters (circERCC6 mutant) restored the miR-412-3p-driven decrease in luciferase activities of circERCC6 reporters (Figure 4D). Thus, it appears to become apparent that circERCC6 sponges miR-412-3p in the SHF stem cells of cashmere goats. 

On the other hand, we noted that the circERCC6 knockdown led to the significant increasing expression of miR-412-3p in SHF stem cells (Figure 4E). However, the miR-412-3p knockdown had no significant effect on the circERCC6 expression level in SHF stem cells of cashmere goats. This suggests that circERCC6 may negatively regulate the miR-412-3p expression level in SHF stem cells. Such regulatory patterns were also reported in previous investigations upon functional analysis of some circRNA molecules, such as circLONP2 in colorectal carcinoma cells [27], as well as circRNA-1926 [9], circRNA-0100 [13], and circRNA-ZNF638 in SHF stem cells of cashmere goats [6].

### 3.5. CircERCC6 Promotes the BNC2 Expression in SHF-Stem Cells of Cashmere Goats through Sponging miR-412-3p

Based on the above obtained results, we found that circERCC6 promoted the induced activation of SHF stem cells of cashmere goats (Figure 2D) in which circERCC6 could directly bind with miR-412-3p (Figure 4B–E). To further define the underlying mechanisms of circERCC6 in contributing to the induced activation of SHF stem cells through the miR-412-3p-mediated pathway, we screened the potential genes of miR-412-3p based on in silico analysis. As a result, a total of 157 potential target genes were predicted with a target score greater than 70. Of them, interestingly, six putative target genes of miR-412-3p were previously revealed to be heavily implicated in regulating the regeneration and development of cashmere goat SHFs including SMAD4, BNC2, FGF12, BMPR1A, SMAD3, and PDGFRA [28]. This drove us to ask whether one or more among these six genes may be involved in the revealed effect of circERCC6 on the induced activation of SHF stem cells via the miR-412-3p pathway. Therefore, we further tested the expression changes in these six genes in circERCC6-knockdown SHF stem cells. We found that only the BNC2 expression was significantly down-regulated in the SHF stem cells after circERCC6 knockdown (Figure 4F) but not for the other tested genes (SMAD4, FGF12, BMPR1A, SMAD3, and PDGFRA). This suggested that circERCC6 might positively regulate the BNC2 expression in SHF stem cells via a miR-412-3p-mediated pattern. 

To confirm this inference, we screened the potential binding sites of miR-412-3p within the BNC2 mRNA 3′-UTR region. As a result, we found a potential binding site of miR-412-3p within the 3′-UTR of the goat BNC mRNA sequence with −27.3 ∆G (Kcal/mol) (Figure 4G). To verify this specific binding between miR-412-3p and the BNC2 mRNA 3′-UTR, a luciferase reporter of BNC2 mRNA 3′-UTR harboring the potential binding site of miR-412-3p was constructed and co-transfected into the SHF-stem cells with si-circERCC6. As observed from Figure 4H, the si-circERCC6-mediated knockdown of circERCC6 in SHF stem cells led to a significant decrease in luciferase activity of BNC2 mRNA in comparison to the counterpart of the siRNA control group. To further confirm this specific interaction between miR-412-3p and BNC2 mRNA 3′-UTR, we constructed a mutation luciferase reporter (BNC2 mRNA 3′-UTR-MUT) containing an antisense mismatch (7 nt long) against the seed region of miR-412-3p (Figure 4I). Subsequently, we co-transfected it into SHF stem cells using miR-412-3p mimics. As recorded in Figure 4J, no significant difference in relative luciferase activity of mutation reporters was observed between miR-412-3p mimics and the control mimic groups. These results confirmed that the miR-412-3p specifically interacts with BNC2 mRNA 3′-UTR. Taken together, a molecular mechanism can be inferred that circERCC6 promotes BNC2 expression in SHF stem cells of cashmere goats through sponging miR-412-3p. 

From a functional point of view, increasing evidence of lines indicated that circRNAs can exert their biological functions in cells via multiple mechanisms, such as miRNA sponging, RNA alternative splicing, gene transcription regulation, RNA maturation, histone modification, protein scaffolding, and protein localization [29]. In this study, our results indicated that circERCC6 served as a molecular sponge of miR-412-3p to enhance BNC2 expression in SHF stem cells of cashmere goats. However, we strongly suggest that the other functional mechanisms of circERCC6 should be further investigated in SHF stem cells, such as its potential roles in regulating the transcription and splicing of the ERCC6 gene, which may have further significance to the physiological process of SHF stem cells in cashmere goats.

### 3.6. The m^6^A Modification Is Required for the circERCC6 Function Exertion via miR-412-3p/BNC2 Pathway

In previous studies, it was demonstrated that m^6^A modifications in various RNAs were essentially involved in the synthesis and metabolism of the RNA molecules, such as the splicing of pre-mRNAs [30], biogenesis initiation of miRNAs [31], and translation initiation of circRNAs [12]. Moreover, it was reported that m^6^A modifications within some non-coding RNA molecules were required for their specific binding with target miRNAs, such as linc1281 [24] and circRNA-ZNF638 [6]. This drives us explore whether m^6^A modification of circERCC6 is required for its effects on the induced activation of SHF stem cells via the miR-412-3p/BNC2 pathway. 

**Figure 5:**
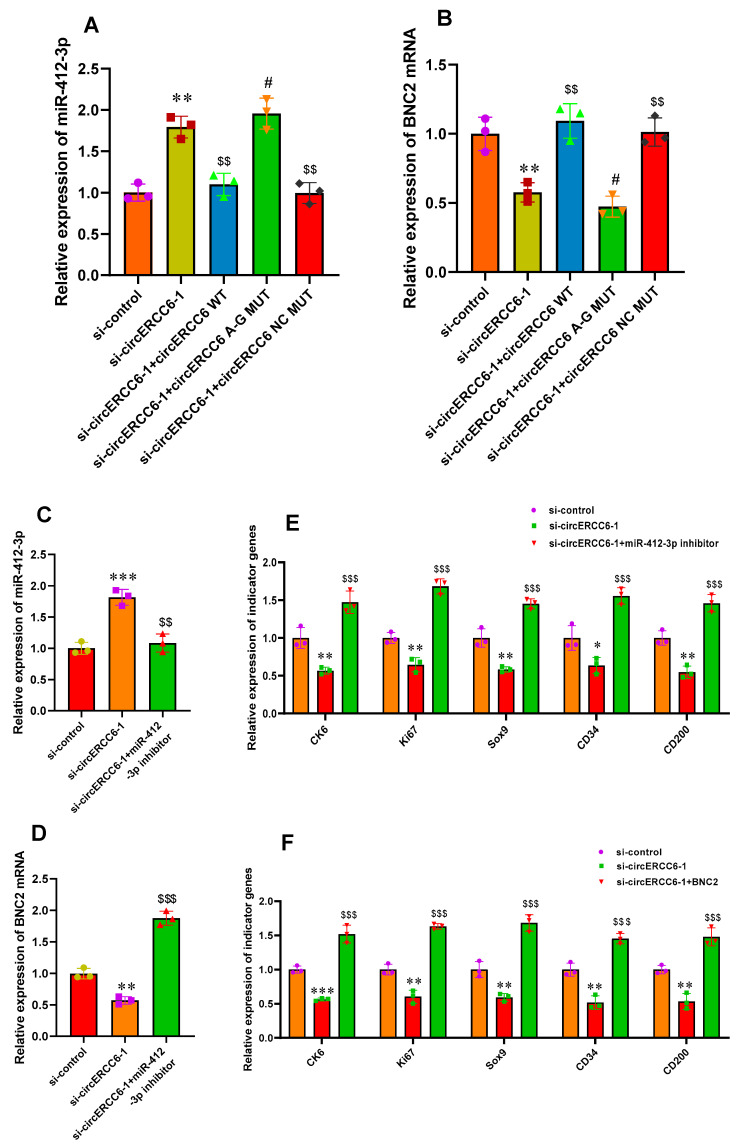
The m^6^A modification is required for circERCC6 function exertion via the miR-412-3p/BNC2 pathway that restored the induced activation of SHF stem cells with circERCC6 deficiency. (**A**) The effects of the mutations of circERCC6 m^6^A modification sites on miR-412-3p expression in SHF stem cells with circERCC6 knockdown. (**B**) The effects of the mutations of circERCC6 m^6^A modification sites on BNC2 expression in SHF-stem cells with circERCC6 knockdown. (**C**) The miR-412-3p inhibitor resulted in significant decreasing expression of miR-412-3p in SHF stem cells with circERCC6 knockdown. (**D**) The miR-412-3p inhibitor resulted in significant increasing expression of BNC2 mRNA in SHF stem cells with circERCC6 knockdown. (**E**) The miR-412-3p inhibitor resulted in significant increased expression of the indictor genes in SHF stem cells with circERCC6 knockdown. (**F**) Overexpression of BNC2 gene resulted in significant increasing expression of the indictor genes in SHF stem cells with circERCC6 knockdown. “*”, “**”, and “***” indicate significant differences in comparison to the si-control group with *p* < 0.05, *p* < 0.01, and *p* < 0.001, respectively. “$$” and “$$$” indicate significant difference in comparison to the si-circERCC6-1 group with *p* < 0.01 and *p* < 0.001, respectively. The “#” indicates no significant difference in comparison to si-circERCC6-1 group with *p* > 0.05.

To verify this inference, we firstly examined the miR-412-3p expression in ‘si-circERCC6-1 + circERCC6 WT’, ‘si-circERCC6-1 + circERCC6 A-G MUT’, and ‘si-circERCC6-1 + circERCC6 NC MUT’ SHF stem cells. As shown in Figure 5A, the ‘si-circERCC6-1 + circERCC6 WT’, and ‘si-circERCC6-1 + circERCC6 NC MUT’ cells had decreasing miR-412-3p expression in comparison to the counterpart of ‘si-circERCC6-1’ cells. Whereas the ‘si-circERCC6-1 + circERCC6 A-G MUT’ cells still had high miR-412-3p expression (Figure 5A), although no significant difference in circERCC6 transcripts exists among these differently treated types of cells (Figure 3C). We further tested the BNC2 mRNA expression in these differently treated types of cells. As a results, we noted that the ‘si-circERCC6-1 + circERCC6 A-G MUT’ cells had decreasing BNC2 mRNA expression in comparison to the counterpart of ‘si-circERCC6-1’ cells (Figure 5B). Whereas, the ‘si-circERCC6-1 + circERCC6 WT’ and ‘si-circERCC6-1 + circERCC6 NC MUT’ cells still had high BNC2 mRNA abundance (Figure 5B). Taken together, these results suggests that m^6^A modification is required for its effects on the induced activation of SHF stem cells via miR-412-3p/BNC2 pathway. 

To date, it is generally thought that the specific binding of circRNAs/lncRNAs with their target miRNAs is driven by a sequence-based base pairing mechanism; however, it remains unclear whether there are certain regulators to mediate this binding between circRNAs/lncRNAs and miRNAs [6,24]. Nevertheless, it was reported that m^6^A peaks at binding sites of mRNAs and miRNAs have suggested a possible mechanism where m^6^A might collaborate with miRNAs to ultimately regulate the target mRNA expression [32]. In this study, our results indicated that m^6^A modification of circERCC6 positively mediated its binding with miR-412-3p, which is confirmed by the m^6^A-deficient A–G mutant of circERCC6 that impeded the binding between circERCC6 and miR-412-3p (Figure 5A,B). Thus, the results from this study suggest a mechanism in which circERCC6 contains both m^6^A modification sites and binding sites of miR-412-3p where its m^6^A modifications collaborate with miR-412-3p to drive the circERCC6-mediated ceRNA regulatory pathway, further promoting the induced activation event of SHF stem cells in cashmere goats. Although it is still unknown whether the m^6^A modifications of circERCC6 change its local structure, as described in previous investigations [6,33], to further facilitate its binding with miR-412-3p, our results provided value information for uncovering m^6^A modification of the circRNA-mediated regulatory mechanism in the induced activation event of SHF stem cells in cashmere goats.

### 3.7. The miR-412-3p/BNC2 Pathway Restores the Induced Activation of SHF Stem Cells with circERCC6 Deficient

Based on above results, we further explore whether the miR-412-3p/BNC2 pathway is responsible for the observed effects of circERCC6 in promoting the induced activation process of SHF stem cells via transfecting miR-412-3p inhibitor in SHF stem cells with circERCC6 knockdown. As a result, the miR-412-3p inhibitor led to a significant decrease in miR-412-3p expression in circERCC6 knockdown cells. (Figure 5C). Concomitant with this, we found that the Wnt5a mRNA level was significantly up-regulated in circERCC6 knockdown cells. (Figure 5D). Moreover, it was recorded that the miR-412-3p inhibition rescued the impaired inducted activation of SHF stem cells with circERCC6 knockdown, which can be defined through the significant increasing expression of the analyzed indicator genes (Figure 5E). 

Meanwhile, as shown above, BNC2 mRNA bound directly with miR-412-3p in its 3′-UTR region (Figure 4G–J) and exhibited decreasing expression in circERCC6-knockdown SHF stem cells (Figure 5B). Thus, we ask whether BNC2 facilitated to the underlying mechanism of circERCC6/miR-412-3p pattern. Therefore, we overexpressed the BNC2 in circERCC6 knockdown SHF stem cells. As a result, we found that the overexpression of BNC2 led to significant increasing expression of the analyzed indicator genes (Figure 5F), which suggests that BNC2 overexpression restores the impaired inducted activation of SHF stem cells resulting from circERCC6 deficiency (Figure 5F). 

**Figure 6:**
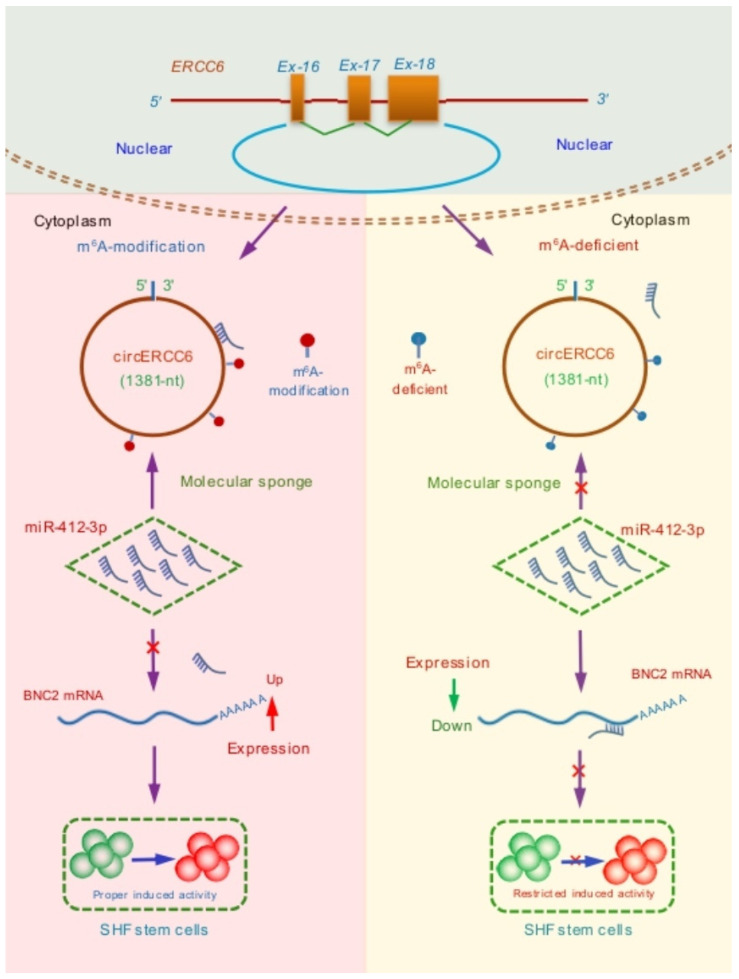
A summary schematic diagram of functional mechanism of circERCC6 in positively regulating the induced activation of SHF stem cells in cashmere goats where m^6^A modification of circERCC6 is required to exert its function through the miR-412-3p/BNC2 pathway.

As is well known, the BNC2, a highly conserved zinc finger protein, is essentially implicated in the regeneration and development of hair follicles. It was recorded that the BNC2 was mainly expressed in the primary hair germs during the development process of hair follicles, and as hair follicle morphogenesis progresses, the cells with BNC2 expression invade the dermis and surround the dermal papilla [34]. Currently, it is unclear whether BNC2 is directly related to the induced activation process of cashmere goat SHF stem cells. However, it is known that the induced activation of hair follicle stem cells requires BMP inhibition [35,36] and Wnt/β-catenin activation [37], whereas the presence of BNC2 is required for both BMP and Wnt/β-catenin signaling pathways in order to lead to the induced activation of stem cells [34]. Here, we confirmed that the BNC2 is ultimately responsible for the circERCC6 effect on the induced activation of SHF stem cells in cashmere goats through the miR-412-3p mediated pathway, where the m^6^A modification of circERCC6 is required (Figure 6). However, it is worth pointing out that we performed the investigation upon the SHF stem cells cultured in vitro. Thus, we strongly suggest that the observed functional significance of circERCC6 with the revealed mechanism should be further verified in SHF stem cells of cashmere goats in vivo. 

## 4. Conclusions

We showed that circERCC6 positively regulates the induced activation of SHF stem cells in cashmere goats, and the m^6^A modification of circERCC6 is required for the exertion of its function via the miR-412-3p/BNC2 pathway. These results would provide a novel and significant treating target for artificially regulating SHF regeneration and cashmere fiber growth per growth cycle to enhance the cashmere yield and quality in cashmere goats. 

## Figures and Tables

**Table 1:** Detail of PCR primers used in present investigation with the corresponding PCR reaction assay and amplicon size.

Gene Name	Reference	Sequence (5′-3′)	Primer Length (nt)	Amplicon Size (bp)	Annealing Temperature (°C)
CircERCC6 (Divergent primers)	Shen et al., 2022 [10]	F: CAGAGACGCCAAGTTTGAGG, R: TGTACACCGTCACCTGCTTT	20	194	55
			20		
*CK6*	Yin et al., 2022 [6]	F: AGTTTGCCTCCTTCATCG, R: GGTTCTGCTTCACGGTCTT	18	111	53
			19		
*Ki67*	Yin et al., 2022 [6]	F: AGGAAGTAGCCAGACTGAGGG, R: GCATCGTGGTTTGCTGTGAA	21	143	56
			20		
*Sox9*	Yin et al., 2022 [6]	F: GGTGCTCAAGGGCTACGACTGG, R: GCGTTGTGCAGGTGCGGGTA	22	162	60
			20		
*CD34*	Yin et al., 2022 [6]	F: GAAGATGTCAGCAGCCACCAG, R: GGCGGTTCATCAGGAAATAGCAC	21	112	56
			23		
*CD200*	Yin et al., 2022 [6]	F: TTGGAAGATGAGGCGTGTTA, R: AGCATTGGCAGAGCAAGTGA	20	156	54
			20		
*GAPDH*	Yin et al., 2022 [6]	F: TGAACCACGAGAAGTATAACAACA, R: GGTCATAAGTCCCTCCACGAT	24	125	53
			21		
CircERCC6 (Me-RIP-qPCR)	This study	F: GGAACTGGTTCAGATGTTCAGAC, R: CTGTCCCCGTTTTCGTGA	23	316	56
			18		
miR-412-3p	MIMAT0036218 in miRNAsong	F: GCACTTCACCTGGTCCACTAGCT	23	Not available	59
*U6*	Han et al., 2020 [18]	F: CGCTTCGGCAGCACATATAC, R:AAATATGGAACGCTTCACGA	20	Not available	55
			20		
*BNC2*	XM_018051926.1	F: CCTCCCGACCAGTCCTATCA, R: CTTCCTGGGCTTCTTCTTGG	20	142	57
			20		

## Data Availability

The data presented in this study are not publicly available and are available on request from the corresponding author.
